# Q Fever, an Under-Recognized and Under-Diagnosed Disease: A Case Report

**DOI:** 10.7759/cureus.70283

**Published:** 2024-09-26

**Authors:** Tommaso Agostino, Megan Edwards

**Affiliations:** 1 Osteopathic Medicine, William Carey University College of Osteopathic Medicine, Hattiesburg, USA; 2 Neurology, Oxford Neurology Clinic, Oxford, USA

**Keywords:** a case report, coxiella burnetii, neurology infectious disease, q-fever, q-fever fatigue syndrome

## Abstract

Q fever is an illness following infection by the organism *Coxiella burnetii*. It is usually associated with an asymptomatic infection in animals which lends a hand in how it is easily transmitted to other hosts such as humans who encounter the bodily fluids of infected animals. Its presentation reflects an acute viral illness with malaise, fever/chills, and vomiting seen in acute cases while endocarditis and other severe sequelae can be seen in chronic infections. Here we have a 55-year-old male who presented for further evaluation of his muscular pains following a motor vehicle accident. His physical exam was benign except for tenderness in his left shoulder and cervical musculature. In interviewing the patient, he reported having fevers/chills, night sweats, and episodes of vomiting for several weeks which led him to seek further medical care. After many tests including a complete blood count (CBC), complete metabolic panel (CMP), blood cultures, and stool/ova testing came back normal, a Q fever phase 1 and 2 titer was performed and confirmed the suspected diagnosis of Q fever due to a lack of his symptoms being explained by another etiology. Although his initial symptoms were consistent with acute Q fever, the continuation of his symptoms suggests the possibility of Q fever fatigue syndrome. This case report highlights the under-recognized, uncommon zoonotic infection that plagues those working in the animal industry while also exploring the various presentations of it.

## Introduction

Q fever is an acute zoonotic illness first discovered in Queensland, Australia in 1935 among meat workers [1]. It is an intracellular pleomorphic bacterium that can infect several hosts ranging from ticks, fish, reptiles, ruminants, and humans. However, sheep and goats are more recognized as reservoir hosts and therefore more critically linked to human infection [2]. The male-to-female ratio is three to one with a positive seroprevalence rate of 3.1% in Americans and is more common among the elderly, Hispanics, and the impoverished [1]. Since the bacteria is mainly transmitted through bodily fluids such as milk, urine, feces, and the placenta of infected animals, it is considered an occupational disease where the risk factors include working with farm animals, consuming raw milk, and slaughtering infected animals.

There are three presentations of Q fever: acute, chronic, and Q fever fatigue syndrome which are recognized with the acute process being the most common. Acute Q fever presents as a self-limiting febrile illness lasting one to three weeks with high fevers (104 F), headaches, fatigue, and myalgias [1]. Our patient presented with these symptoms in addition to suffering from a tractor wreck in which he was ejected from the tractor. This case report highlights this illness as a rare but important diagnosis and delves into some of the symptoms of the varying types of presentations. This case report was previously presented as an abstract poster presentation at the 2024 American Academy of Neurology (AAN) Summer Conference on July 19, 2024.

## Case presentation

Patient information

A 55-year-old male presented to the Oxford Neurology Clinic for pain and stiffness in his shoulders and neck. During this visit, the patient stated he was involved in a tractor wreck in August 2023 but continued to have joint pains, recurrent fevers, and malaise for several weeks afterward; he owns a farm and was carrying a bale of hay when he was hit. He visited several physicians and had routine testing in addition to C. difficile toxin A and B, and pancreatic fecal elastase lab tests which did not reveal any abnormalities. With the addition of night sweats and the constant feeling of having the flu, he finally was referred to an infectious disease physician who diagnosed him with Q fever the following October after conducting a variety of laboratory tests including Q fever Phase 1 and 2 titers. His risk factors for Q fever stem from his work on his farm and interactions with the animals on them, but it is unclear if he was exposed to any bodily fluids in the recent past. He has a past medical history of diabetes, hemochromatosis, and avascular necrosis.

Clinical findings and diagnostic assessment

On examination, the patient was in no acute distress but reported muscle and joint tenderness with palpation of his left shoulder, upper back, and neck musculature. The sensation of light touch and muscle strength were both intact.

At the time of his presentation to the emergency room (ER) after his motor vehicle accident, he received imaging consisting of a CT chest abdomen/pelvis w contrast, CT head wo contrast, CT facial bones wo contrast, CT cervical spine WO contrast, and a chest and pelvis x-ray that were all negative for fractures, bleeding, or any other abnormalities. With the addition of his recurrent fevers, chills, and vomiting for weeks following his accident, differential diagnoses included parasitic infections, sequelae from his chronic diseases, and trauma from his motor vehicle accident which were ruled out by performing routine metabolic and infectious workups, which included a complete blood count (CBC), complete metabolic panel (CMP), viral and bacterial panels and a C diff toxin test, along with a bone marrow biopsy and ova and parasite testing. Due to the lack of explanation for his symptoms, relevant laboratory testing being negative and no improvement in his symptoms, a more chronic process was thought to be involved, and an infectious disease physician was recruited to investigate further. A diagnosis of Q fever could be a plausible explanation for his symptoms because it presents similarly to a flu-like illness with symptoms the patient reported such as malaise, fever/chills, and body aches in the absence of improvement with treatment. Additional testing was performed with a Q fever IgM phase 1 titer of 1:16 and a Q fever IgG phase 2 titer of 1:64. 

The diagnosis was made primarily from his positive Q fever phase titers but also the results of his other pertinent labs such as an elevated fecal calprotectin of 83 (borderline elevated > 50-120), and normal pancreatic fecal elastase of 330 (normal > 200) suggested that there was some kind of inflammatory process occurring in addition to his acute motor vehicle accident (MVA) that could not be localized. Moreover, his symptoms of recurring fevers, arthralgias, malaise, and night sweats strongly suggested Q fever over other diagnoses. Differentiating the presentations of Q fever - acute, chronic, and Q fever fatigue syndrome - is mainly done by the presenting symptoms and onset/chronicity [1]. Acute Q fever is usually seen with one to three weeks of milder symptoms that self-resolve whereas chronic Q fever is seen with more severe presentations and is more deadly. Q fever fatigue syndrome is a newer presentation seen in individuals whose presentation is more focused on the fatigue component. Further elaboration on these presentations will be done in the discussion section.

Therapeutic intervention

The patient was started on oral ciprofloxacin and doxycycline. He also received a pic line and was administered IV doxycycline for 14 days.

Follow-up and outcomes

The patient is currently on oral doxycycline and is following up regularly with his infectious disease doctor. He did not report any side effects but does complain of some lingering malaise and flu-like symptoms. He must still be monitored for any recurrence of fevers and fatigue, as some of the more chronic presentations can present with these symptoms and may be more refractory to treatment.

## Discussion

This patient’s presentation of Q fever was very similar to the classic manifestations of mild flu symptoms, sweating, intermittent fevers, and gastrointestinal tract (GIT) involvement but his continuation to feel symptoms while on appropriate antibiotics coupled with his comment of “feeling like having a persistent flu” suggests that our patient is manifesting what is known as Q fever fatigue syndrome [2]. Q fever fatigue syndrome (QFS) is a debilitating condition that occurs in approximately 20% of patients and can have effects on a patient’s social and economic livelihood with QFS being thought to have been associated with the financial losses of the Dutch Q fever outbreak of 2007-2010 [2]. Some of the symptoms seen in QFS include persistence of fatigue over six months, headaches, blurring of vision, and impaired memory although our patient either did not display all of these or was not asked at the time of interview [3]. Chronic Q fever is another syndrome that can occur which leads to sequelae such as endocarditis, osteomyelitis, nephritis, etc. which our patient did not display. Some research into the pathophysiology of QFS has shown that there are mitochondrial-derived peptide-coding genes that may be playing a role in the fatigue component of the disease; however, more research needs to be conducted [4]. Specifically, there are two mitochondrial-derived peptides mentioned in the literature, humanin and mitochondrial ORF of the 12S rRNA type-C (MOTS-c), that are involved in mitochondrial dysfunction in circulating monocytes in affected patients. Further research could involve discovering if gene therapies or exogenic administration of these peptides could benefit these patients and potentially reverse the condition. Moreover, the pathophysiology of Q fever itself has not been very well investigated and is thought to include invasion and replication within the lysosomal vacuoles in addition to having variation in its lipopolysaccharide (LPS) [2]; research into potentially developing techniques to inhibit the replication and invasion of these lysosomal vacuoles could be beneficial in preventing infection.

## Conclusions

Our case’s presentation of Q fever alone warrants the healthcare community to increase their awareness of this disease as its crucial connection to the animal and food industry makes it an undeniable source of infection not to mention the bioterrorism potential if the wrong person(s) got a hold of the organism responsible. Since those who are impoverished are of minority status, and those who directly work with animals are more at risk of infection, it is important to realize this and take action in the form of creating more strict protocols for the handling of animals and related products, such as milk and meats, as well as providing education to families about Q fever and how it can be transmitted. Communication about disease transmission and prevention is the backbone of a well-developed plan of action to halt the spread of this disease. Furthermore, more research into the complications and sequelae of Q fever is one of the next steps as the already limited information on the disease makes it more difficult to properly treat these patients. If the world was more aware of Q fever and did not disregard it as a rare illness seen in textbooks or developing nations, then we believe that this would lead to the possibility of new developments in the treatments of the less common manifestations such as chronic Q fever and QFS.
